# Comparative analysis of adhesion virulence protein FadA from gut-associated bacteria of colorectal cancer patients (*F. nucleatum*) and healthy individuals (*E. cloacae*)

**DOI:** 10.7150/jca.98951

**Published:** 2024-08-19

**Authors:** Nadia Hussain, Fatima Muccee, Naeem Mahmood Ashraf, Tayyaba Afsar, Fohad Mabood Husain, Arslan Hamid, Suhail Razak

**Affiliations:** 1 Department of Pharmaceutical Sciences, College of Pharmacy, Al Ain University, Al Ain Campus, Al Ain 64141, Abu Dhabi, United Arab Emirates.; 2 AAU Health and Biomedical Research Center, Al Ain University, Abu Dhabi Campus, Abu Dhabi P. O. Box 112612, Abu Dhabi, United Arab Emirates.; 3 School of Biochemistry and Biotechnology, University of Punjab, Lahore, 52254, Pakistan.; 4 Department of Community Health Sciences, College of Applied Medical Sciences, King Saud University, Riyadh, Saudi Arabia.; 5 Department of Food Science and Nutrition, College of Food and Agriculture Sciences, King Saud University, Riyadh, Saudi Arabia.; 6 University of Bonn, LIMES Institute (AG-Netea), Carl-Troll-Str. 31, 53115 Bonn, Germany.

**Keywords:** Colorectal cancer, *F. nucleatum*, *E. cloacae*, FadA virulence factor

## Abstract

**Background:** Colorectal cancer (CRC) is a gastrointestinal disease linked with GIT microbial dysbiosis. The present study has targeted the comparative analysis of virulent factor FadA from gut-associated bacteria of CRC patients (*F. nucleatum*) and healthy individuals (*E. cloacae*).

**Methods:** For this purpose, FadA protein sequences of fifteen strains of *F. nucleatum* and four strains of *E. cloacae*, were retrieved from the UniProt database. These sequences were analysed through VirulentPred, PSLpred, ProtParam, PFP-FunDSeqE, PROTEUS Structure Prediction Server, SWISS-MODEL, SAVES validation server, MEME suite 5.5.0, CAVER Web tool, Webserver VaxinPAD, HPEPDOCK and HDOCK servers.

**Results:** FadA protein from *F. nucleatum* was found to exhibit significant differences as compared to *E. nucleatum* i.e. it exhibited helical configuration, cytoplasmic, periplasmic, outer-membrane and extracellular localisation, 2D structure comprising of 70-96% helix, 0% beta-sheet, 4-30% coils and 17-20 signal peptide residues, hydrophilicity, strongly acidic character and smaller number of antigenic epitopes. In contrast, FadA protein from *E. nucleatum* was found to have globular 3D configuration, cytoplasmic localisation, 2D structure (30-56% helix, 12-21% beta-sheet, 33-50% coils and 43 signal peptide residues), highly hydrophobic, slightly acidic and more number of antigenic epitopes. Docking analyses of virulent factors revealed their high binding affinities with previously reported inhibitory peptide and FAD-approved drug COX2.

**Conclusion:** The wide range of differences not only provided us the reason for the role of FadA protein as a virulent factor in *F. nucleatum* but also might help us in designing virulent FadA protein inhibiting strategies including peptide-based vaccine adjuvants and drugs designing, modification of tunnels and catalytic pockets to reduce substrate binding and FAD approved drugs selection. Inhibition of this virulent factor in CRC patients' gut bacteria might result in oncogenesis regression and reduced death rate.

## Background

Colorectal cancer (CRC) is the second and third most abundant cancer concerning morbidity (9.2%) and diagnosis (6.1%) as per the research of the Centers for Disease Control and Prevention (CDC). According to a study, by 2030 CRC is expected to increase by 60% with 2.2 million new cases and 1.1 million deaths 1. It has contributed to 9, 35000 deaths caused by cancer. It is characterized by polyps in the rectum, change in bowel habits, constipation, diarrhoea, rectal bleeding, change in stool colour, shape, abdominal cramps, gas, pain, changed stool consistency, tenesmus, anaemia, loss of appetite and weight, nausea, vomiting, jaundice and fatigue 1. The risk factors include smoking, physical inactivity, obesity, unhealthy and improper diet routine, family history of diabetes, inflammatory bowel diseases, colon polyps, genetics, race, gender, age and gastrointestinal tract (GIT) microbiome 2, 3.

The composition of the GIT-associated microbial population always varies with oncogenesis. This dysbiosis always involves a shift toward pathogenic microbes including *Fusobacterium nucleatum*, *Escherichia coli*, *Enterococcus faecalis*, *Bacteroides fragilis*, *Salmonella enterica*, *Streptococcus*, *Rothia*, *Faecalibacterium*, *Porphyromonas*, *Collinsella*, *Slackia*, *Alistipes*, *Porphyromonas*, *Prevotella*, *Methanobrevibacter*, *Gemella*, *Mogibacterium*, *Parvimonas*, *Peptostreptococcus*, *Solobacterium*, *Helicobacter*, *Klebsiella*, *Akkermansia* and *Thermanaerovibrio* 4-14. Following bacteria have been reported to reduce in CRC patients i.e. *Ruminococcus*, *Roseburia*, *Lactobacillus*, *Eubacterium*, *Clostridium*, *Anaerostipes*, *Bacteroides*, *Bifidobacterium*, *Citrobacter*, *Treponema*, *Serratia*, *Kluyvera* and *Cronobacter* 10-20.

*E. cloacae* is reported as a harmless GIT commensal microbe normally found in healthy individuals 21. On the contrary, *F. nucleatum* is a commensal-turned and obligate pathogen associated with various GIT disorders 22. It potentiates the development and progression of CRC due to its enrichment in colorectal malignant tumour tissue 23-25. Being asaccharolytic, it metabolizes the peptides and amino acids into short-chain fatty acids and formyl-methionyl-leucyl-phenylalanine 26. These metabolites attract myeloid cells in the tumour microenvironment thus contributing to tumour enrichment with myeloid cells. Additionally, metabolic products also promote vascularization and infiltration of immune cells in tumours 9. Being slightly capable of respiring aerobically, *Fusobacterium* successfully survives hypoxic tumour conditions 27. It increases oncogenic micro RNAs 28-30. It damages DNA and induces single nucleotide polymorphisms (SNPs) by producing reactive oxygen species via its metabolite hydrogen sulfide 31. It suppresses tumour immune microenvironment via interference with functions of T-cells, macrophages, dendritic cells, neutrophils and natural killer cells (NKCs) 9, 32. Therefore, it can be considered one of the major non-invasive prognostic, diagnostic and therapy response biomarkers of CRC 9, 33-42.

*F. nucleatum* being an adhesive pathogen has multiple virulence factors contributing to its colonization of colorectal tissues and CRC incidence. The known virulence factors of *F. nucleatum* promote adhesion to intestinal epithelial cells via FadA 8. This factor has a strong connection with CRC due to its association with NF-Kb inflammatory response and cell adhesion and invasion of *Fusobacterium* 43. It enables the bacterium to get attached to fibroblasts, and endothelial and epithelial cells 22. To cause disease, FadA binds the EC5 domain (ASANWTLOYNDP) of the E-cadherin protein leading to its phosphorylation. This activates the Wnt signal in the Wnt/β- cadherin pathway. Wnt binds receptors and disrupts the destruction complex comprising of Axin, APC, GSK-3β, PP2A and Ck1α. This complex under normal circumstances targets β-catenin for ubiquitination and digestion by proteasome. Disruption of this complex inhibits phosphorylation of β-catenin resulting in its stability. The β-catenin gets accumulated in the cytosol and translocated to the nucleus. In the nucleus, β-catenin binds with TCF/LEF transcription factors and activates the transcription of genes involved in inflammation and other cancer-initiating activities 44.

The present study focused on comparative analysis of the FadA protein in *F. nucleatum* and *E. cloacae* at the levels of physicochemical properties, sub-cellular localization, conserved domains, antigenic epitopes, functional domains, 2D and 3D structures, the number of catalytic sites and tunnels that might help design strategies for inhibition of tumorigenic roles of *F. nucleatum* FadA protein.

## Methodology

### Retrieving the sequences of FadA protein in *F. nucleatum* and *E. cloacae*

Sequences of FadA protein in *F. nucleatum* and *E. cloacae* were retrieved from the UniProt database (https://www.uniprot.org, accessed on 11 Nov. 2022) 18. Sequences were retrieved for a total of fifteen strains of *F. nucleatum* and four strains of *E. cloacae* (Supplementary data Table 1).

### Evaluation of virulence potential

To predict the virulence status of the FadA protein in bacteria documented in the present study VirulentPred tool (http://bioinfo.icgeb.res.in/virulent/, accessed on 13 Nov. 2022) has been consulted 45. This is a Support Vector Machine (SVM) based method. It was determined based on prediction scores. A positive score indicates virulence while a negative score shows the non-virulent nature of protein.

### Phylogenetic tree construction

To analyze the phylogenetic relationship among present study bacteria, the Clustal Omega Multiple Sequence Alignment Tool was used for multiple sequence alignment 46. Alignment was followed by tree construction using MEGA version 7. Trees were inferred by the neighbour-joining method 47, 48. The evolutionary distances were computed using the Poisson correction method and are in the units of the number of amino acid substitutions per site 49.

### Evaluation of sub-cellular localization

To predict the sub-cellular localization of proteins, the PSLpred tool (webs.iiitd.edu.in/raghava/pslpred/submit.html, accessed on 26 Nov. 2022) was used 50. The prediction approach used was based on a hybrid approach.

### Evaluation of physicochemical properties

To determine the differences between FadA proteins of bacteria documented in the present study, the ProtParam tool (https://web.expasy.org/protparam/, accessed on 12 Dec. 2022) was used 51. Properties compared include the number of amino acids, molecular weight, theoretical isoelectric point (pI), half-life, instability index, aliphatic index, extinction coefficient and grand average of hydropathy (GRAVY) 51. The pI value helps in prediction of the acidity or alkalinity of the protein.

### Prediction of functional domains

To predict the functional domains in FadA protein sequences of bacteria inhabiting normal and cancerous patients GIT, Functional domain or motif prediction (PFP-FunDSeqE) tool (www.csbio.sjtu.edu.cn/bioinf/PFP-FunDSeqE/#, accessed on 25 Nov. 2022) was used 52.

### Prediction of 2D configuration

To predict the 2D configuration of the present study proteins, PROTEUS Structure Prediction Server 2.0 (www.proteus2.ca/proteus2/, accessed on 20 Dec. 2022) was used 53. This server helped us to determine the helix, beta sheet, coil content, signal peptide and membrane content of proteins 54.

### Prediction of 3D configuration

To predict the 3D structure of bacterial proteins, a homology modelling server SWISS-MODEL (https://swissmodel.expasy.org, accessed on 12 Dec. 2022) was used 55.

### Validation of 3D structures

To validate the 3D structures of FadA proteins predicted using the SWISS-MODEL, the SAVES validation server (https://saves.mbi.ucla.edu, accessed on 20 Dec. 2022) was used. Two programs of this server i.e. ERRAT and PROCHECK were used.

### Prediction of conserved protein motifs

To predict the conserved protein motifs, Multiple Em for Motif Elicitation (MEME) suite 5.5.0 (https://meme.suite.org/meme/tools/meme, accessed on 21 Dec 2022) was used. A total of ten motifs were found using default values of all parameters. For ontology analysis of predicted motifs, Lambda Predict Protein (ƛPP) (https://embed.predictprotein.org/0, accessed on 23 Dec 2022) was used.

### Assessment of catalytic pockets and tunnels

To assess the catalytic pockets and tunnels in FadA proteins of bacteria documented in the present study, the CAVER Web tool was used (https://loschmidt.chemi.muni.cz/caverweb, accessed on 20-21 Dec. 2022) 56.

### Evaluation of immunomodulatory A-cell epitopes

Webserver VaxinPAD (https://webs.iiitd.edu.in/raghava/vaxinpad/batch.php, accessed on 25 - 27 Dec 2022) was used to explore the immunomodulatory A-cell epitopes in the FadA protein of *F. nucleatum* and* E. cloacae* 57.

### Assessment of binding affinity of FadA with inhibitor peptide

To determine the binding affinity of FadA protein from virulent *F. nucleatum* bacteria with a previously reported inhibitor peptide ASANWTIQYND, peptide-protein docking web-server, HPEPDOCK (huanglab.phys.hust.edu.cn/hpepdock/, accessed on 26 & 27 Dec. 2022) was used 8, 58. To dock FadA protein with cyclooxygenase-2 (COX2), the HDOCK server (http://hdock.phys.hust.edu.cn/, accessed on 31 Dec, 2022) was used 59.

## Results

### Virulence status prediction

Sequences of the FadA protein retrieved from the UniProt database were analyzed for virulence status. All the sequences from *E. cloacae* were found to be non-virulent with negative scores while those from *F. nucleatum* showed virulent status with positive scores (Table 1).

### Phylogeny

The optimal tree for the *F. nucleatum* strains with the sum of branch length 11.14917307 is shown in Supplementary Data Figure 1 (a). According to this tree, *F. nucleatum* IV and V, *F. nucleatum* II and III, *F. nucleatum* I and subsp. *polymorphum* 3 and *F. nucleatum* subsp. *animalis* 7_1 and *animalis* D11 (I) were closely related as compared to others as they share the same clade with each other.

The optimal tree for the *E. cloacae* strains with the sum of branch length 3.79674773 is shown in Supplementary Data Figure 1 (b). According to this tree, *E. cloacae* I and IV are more closely related to each other as compared to others as they are originating from the same branch point. While *E. cloacae* III and II are distantly related to each other and from *E. cloacae* I and IV.

### Sub-cellular localization prediction

Assessment of sub-cellular localization of FadA protein using PSLPred analysis tool revealed FadA protein to be localized in the cytoplasm in the case of all four strains of *E. cloacae*. in the case of colorectal cancer GIT bacteria, in addition to the cytoplasm (*F. nucleatum* (IV), *F. nucleatum* (VI), *F. nucleatum* 13_3C, *F. nucleatum* CTI-5, *F. nucleatum* subsp. *Vincentii*, *F. nucleatum* subsp. *animalis* 7_1, *F. nucleatum* subsp. *animalis* D11 (I), *F. nucleatum* subsp. *animalis* D11 (II) and *polymorphum* 2) the protein was also found to be localized in periplasmic space (*F. nucleatum* (II) and *F. nucleatum* (III)), outer-membrane (*F. nucleatum* (I) and *polymorphum* 3) and extracellular membrane (*F. nucleatum* (V) and *F. nucleatum* subsp. *animalis* 11_3_2) (Supplementary data Table 2).

### Physicochemical properties prediction

Analysis of the physicochemical properties of FadA proteins revealed a wide range of variations in proteins of *F. nucleatum* from *E. cloacae*. In *E. cloacae*, the pI was observed in the range of 5.97 to 6.90 while in the case of *F. nucleatum*, the minimum and highest values of pI were observed to be 4.06 and 6.94, respectively. The highest deviation was observed in the case of *F. nucleatum* I, *F. nucleatum* II, *F. nucleatum* III, *F. nucleatum* IV, *F. nucleatum* subsp. *polymorphum* 2 and *F. nucleatum* subsp. *polymorphum* 3 (Table 2). *F. nucleatum* II, *F. nucleatum* III and *F. nucleatum* IV showed a very unusual value of half-life i.e. 2 min., as compared to all other bacteria documented in the present study. High deviation of instability index from *E. cloacae*, was observed in the case of *F. nucleatum* II (40.2), *F. nucleatum* V (46.80), *F. nucleatum* VI (48.33), *F. nucleatum* 13_3C (52.95), *F. nucleatum* CTI-5 (49.32), *F. nucleatum* subsp. *vincentii* (51.94), *F. nucleatum* subsp. *animalis* 7_1 (47.92), *F. nucleatum* subsp. *animalis* D11 I (50.36), *F. nucleatum* subsp. *animalis* D11 II (50.37), *F. nucleatum* subsp. *animalis* 11_3_2 (46.13), *F. nucleatum* subsp. *polymorphum* 2 (43.71) and *F. nucleatum* subsp. *polymorphum* 3 (69.24). As far as the aliphatic index is concerned, *F. nucleatum* I (82.79), *F. nucleatum* II (77.45), *F. nucleatum* III and *F. nucleatum* IV (75.79), *F. nucleatum* V (81.41) and *F. nucleatum* subsp. *polymorphum* 3 (75.32) exhibited variation from that of *E. cloacae*. *F. nucleatum* III and *F. nucleatum* IV (2980), *F. nucleatum* V and *F. nucleatum* subsp. *animalis* 11_3_2 (11460), *F. nucleatum* subsp. *animalis* D11 II (12950), *polymorphum* 2 (4470) and *polymorphum* 3 (1490) was observed to exhibit variation in extinction coefficient values than the GIT inhabiting bacteria from healthy individuals. All the bacteria from CRC patients' gut were found to have very different values of GRAVY i.e. ranging from -0.440 to -1.460 as compared to bacteria from healthy individuals (Table 2).

### Functional domains prediction

Functional domain analyses also revealed a large degree of variation of FadA proteins between *F. nucleatum* and *E. cloacae* strains. Two fold types were found in *E. cloacae* protein i.e. NAD(P)-binding Rossmann-fold and (TIM)-barrel. On the other hand, four different types of fold were observed in *F. nucleatum* strains i.e. 4 helical up and down bundle, DNA-binding 3-helical bundle, EF-hand and 4-helical cytokines (Table 3).

### Two-dimensional structures prediction

The 2D structure of *F. nucleatum* FadA protein was markedly different from that of *E. cloacae*. i. e. the α-helix content was significantly higher in *F. nucleatum* (70-96%) as compared to *E. cloacae* (30-56%). No beta sheets were observed in *F. nucleatum* while *E. cloacae* secondary structure exhibited 12-21% beata sheets. Coil content was extremely lower in *F. nucleatum* (4-16%) than *E. cloacae* (33-50%). Only *E. cloacae* III exhibited signal peptide (17%) content while the majority of the *F. nucleatum* strains were found to have signal peptide content i.e. 14-19% (Table 4, Supplementary data Figure 2).

### Three-dimensional structures prediction

**Figure 1:**
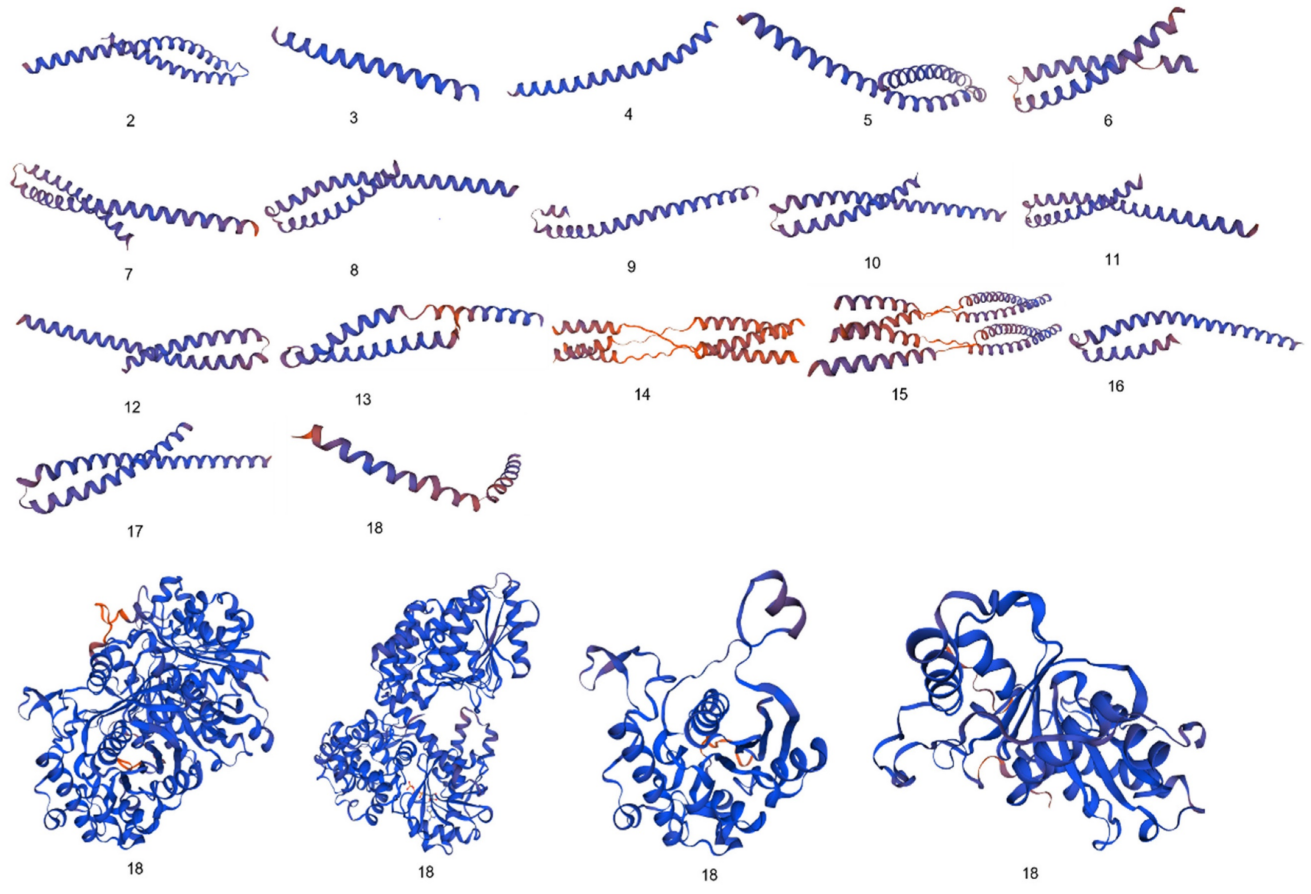
Comparative analysis of 3D configuration of adhesion virulence factor FadA between the *F. nucleatum* strains belonging to colorectal cancer patients and *E. cloacae* strains belonging to healthy individuals. a: *F. nucleatum* (I), b: *F. nucleatum* (II), c: *F. nucleatum* (III), d: *F. nucleatum* (IV), e: *F. nucleatum* (V), f: *F. nucleatum* (VI), g: *F. nucleatum* 13_3C, h: *F. nucleatum* CTI-5, i: *F. nucleatum* subsp. *Vincentii*, j: *F. nucleatum* subsp. *animalis* 7_1, k: *F. nucleatum* subsp. *animalis* D11 (I), l: *F. nucleatum* subsp. *animalis* D11 (II), m: *F. nucleatum* subsp. *animalis* 11_3_2, n: *F. nucleatum* subsp. *polymorphum* 1, o: *F. nucleatum* subsp. *polymorphum* 1, p: Enterobacter cloacae (I), q: Enterobacter cloacae (II), r: Enterobacter cloacae (III), s: Enterobacter cloacae (IV).

Significant diversity was found between the FadA protein of *F. nucleatum* and *E. cloacae* at the level of 3D configuration as is evident from Figure 1. The globular structure was found in *E. cloacae* proteins while only a helical folding pattern was observed in the case of *F. nucleatum*. Validation of these structures using Procheck and ERRAT scores is described in detail in Supplementary Data Table 3.

### Catalytic pockets and tunnels prediction

**Figure 2:**
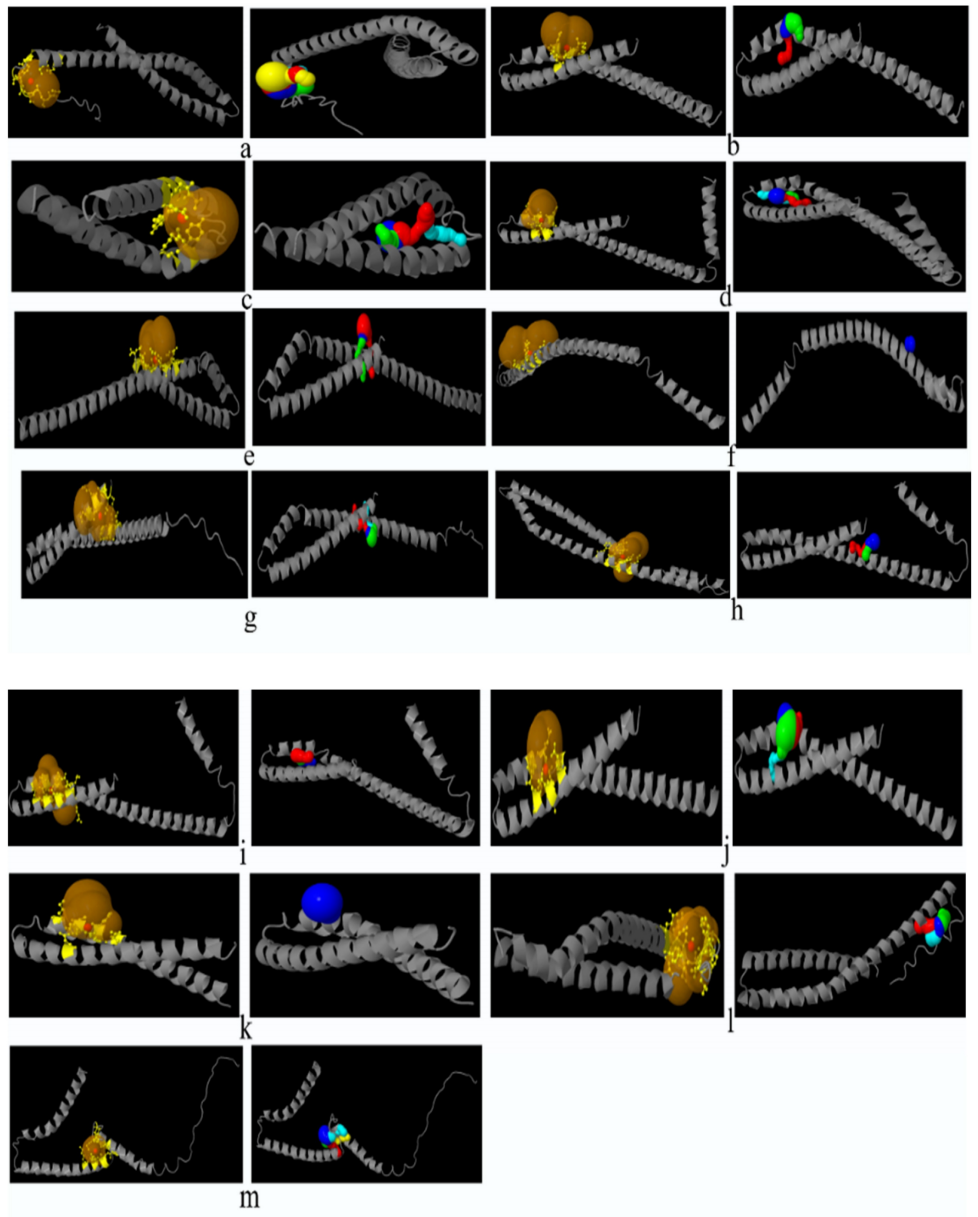

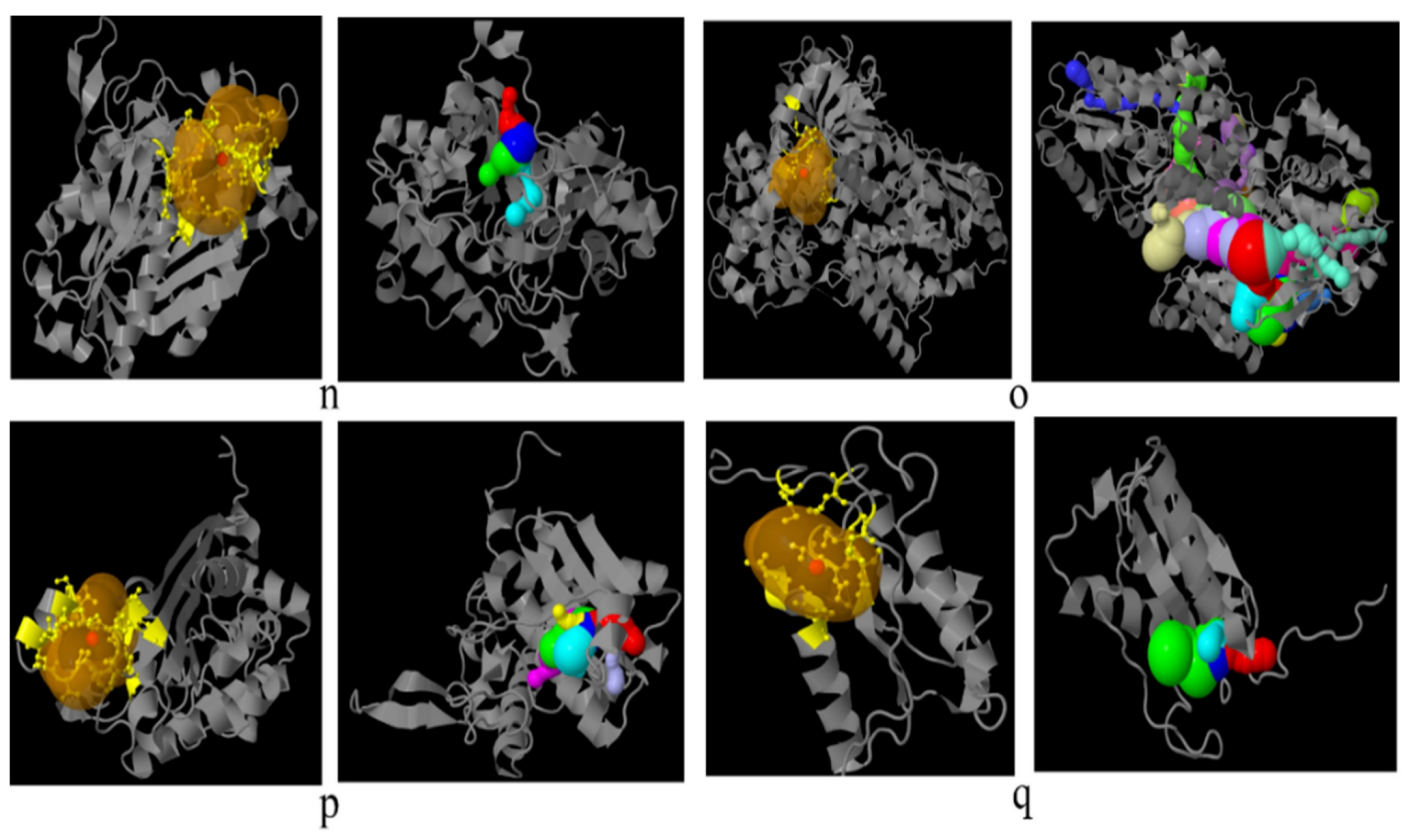
Prediction of catalytic pockets and tunnels using CAVER Web tool in bacteria documented in present study. a: *F. nucleatum* (I), b: *F. nucleatum* (IV), c: *F. nucleatum* (V), d: *F. nucleatum* (VI), e: *F. nucleatum* 13_3C, f: *F. nucleatum* CTI-5, g: *F. nucleatum* subsp. *Vincentii*, h: *F. nucleatum* subsp. *animalis* 7_1, i: *F. nucleatum* subsp. *animalis* D11 (I), j: *F. nucleatum* subsp. *animalis* D11 (II), k: *F. nucleatum* subsp. *animalis* 11_3_2, l: *F. nucleatum* subsp. *polymorphum* 1, m: *F. nucleatum* subsp. *polymorphum* 1, n: Enterobacter cloacae (I), o: Enterobacter cloacae (II), p: Enterobacter cloacae (III), q: Enterobacter cloacae (IV), yellow part: catalytic pocket, multiple colors region = multiple tunnels.

All the bacteria except *F. nucleatum* II and *F. nucleatum* III were found to exhibit catalytic pockets and tunnels. The highest number of tunnels was observed in *E. cloacae* II (Table 5, Figure 2).

### Prediction of conserved motifs

**Figure 3:**
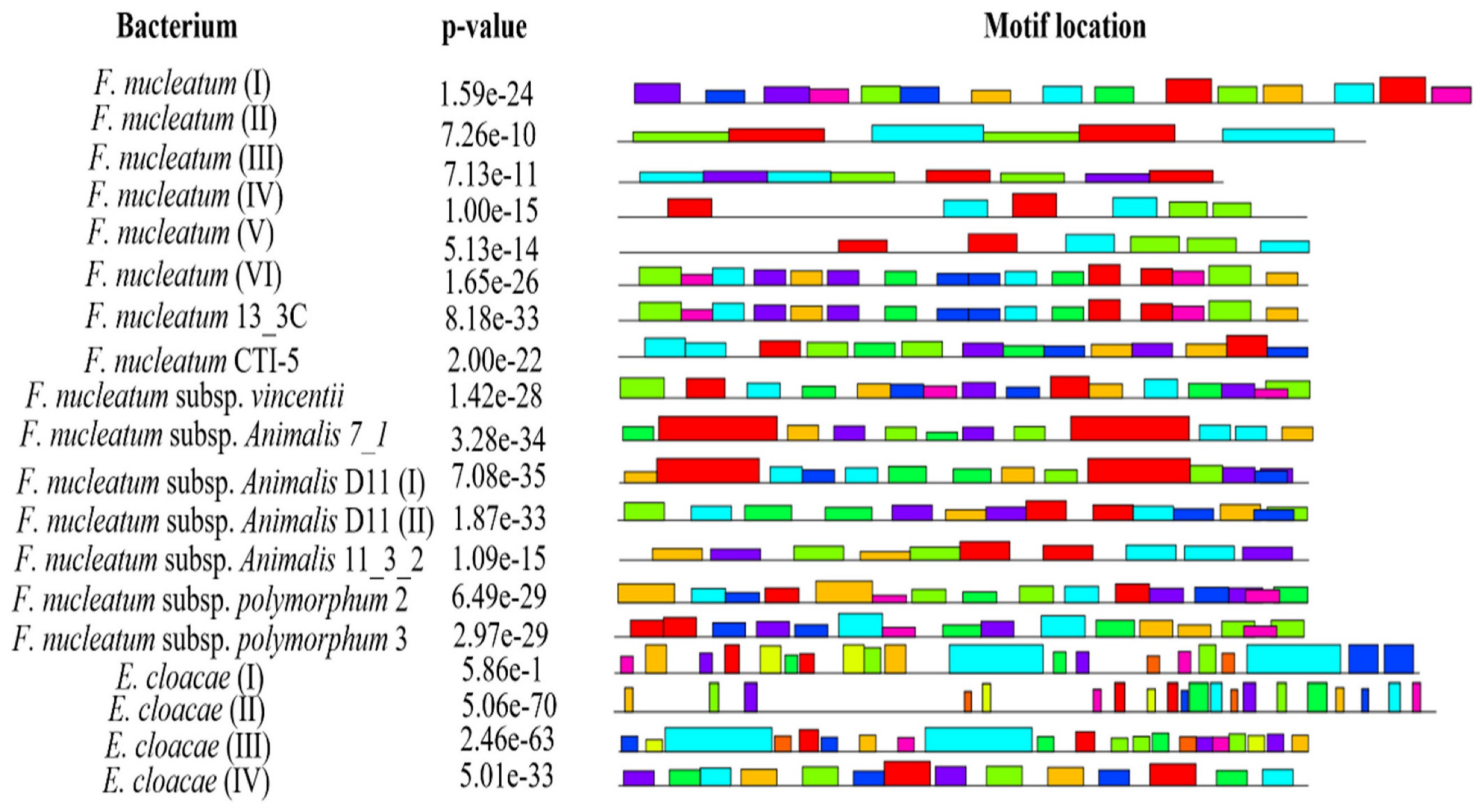
Location of conserved motifs with corresponding combined match p-value for FadA protein of *F. nucleatum* and *E. cloacae*, predicted using MEME suite. Each colored block is depicting strength and position of each motif site. Height of block is directly proportional to significance of predicted site. Different motif sites are represented using different colors.

Conserved motif analysis revealed that all the bacteria documented in the present study except *F. nucleatum* II, *F. nucleatum* III, *F. nucleatum* IV and *F. nucleatum* V exhibited the conserved regions (Figure 3, Supplementary data Table 4).

### A-cell epitopes prediction

Epitope assessment revealed that a sufficient number of antigenic peptides were present in FadA protein from healthy individuals' GIT. i.e. *E. cloacae* I (52), II (40), III (28) and IV (18). However, in most of the bacteria from CRC patients GIT, FadA was found to have very few epitopes like *F. nucleatum* subsp. *polymorphum* 2 (1), *F. nucleatum* subsp. *polymorphum* 3 (6) and *F. nucleatum* I (4) or no epitopes i.e. *F. nucleatum* II and III. Others were found to have epitopes in the range of 12-20 (Supplementary data Table 5).

### Docking of FadA protein inhibitor peptide

**Figure 4:**
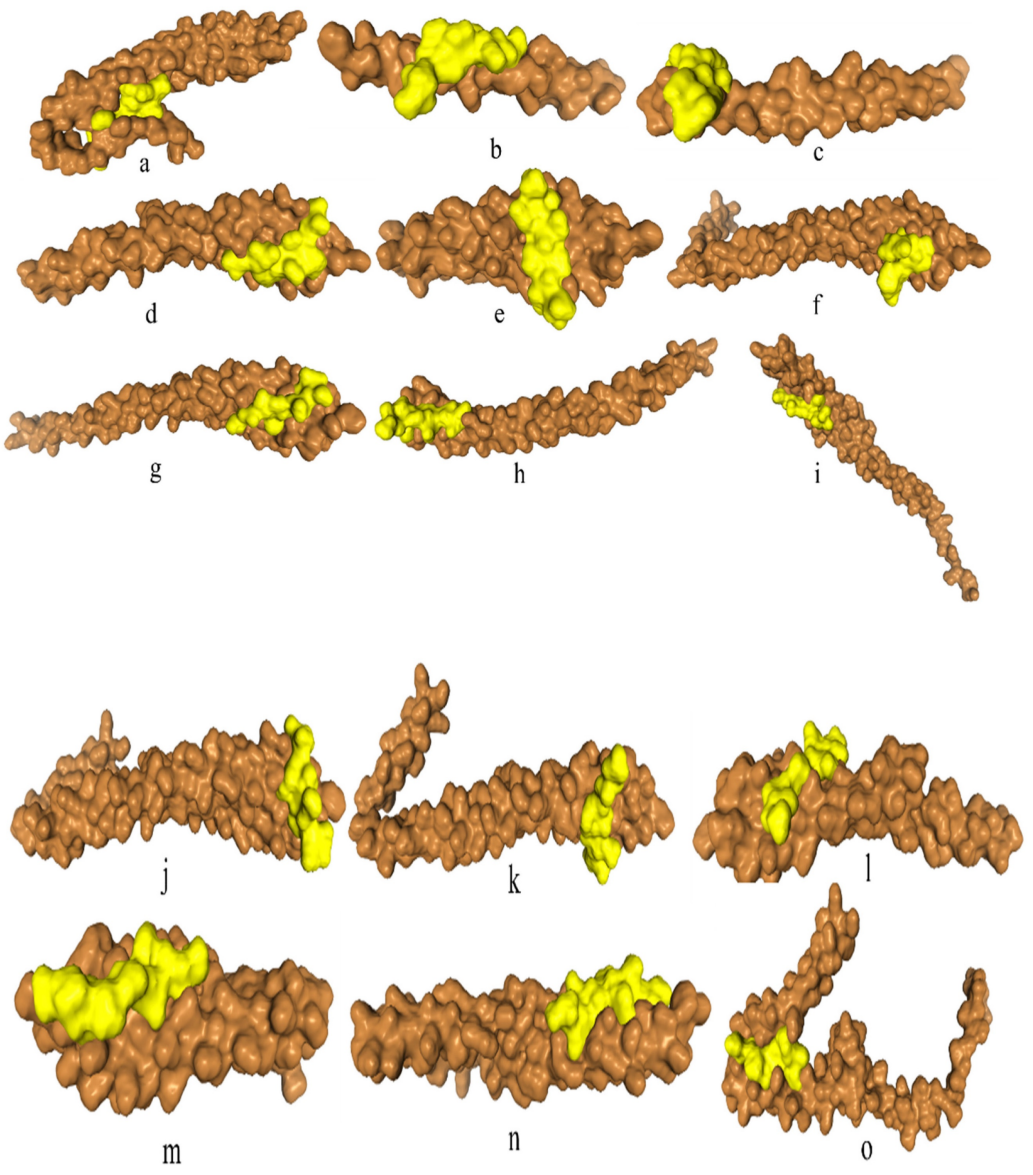
Docking of FadA virulence factor from different strains of *F. nucleatum* with inhibitory peptide ASANWTIQYND reported in literature. FadA protein and inhibitory peptide are represented in brown and yellow colors, respectively. a: *F. nucleatum* (I), b: *F. nucleatum* (II), c: *F. nucleatum* (III), d: *F. nucleatum* (IV), e: *F. nucleatum* (V), f: *F. nucleatum* (VI), g: *F. nucleatum* 13_3C, h: *F. nucleatum* CTI-5, i: *F. nucleatum* subsp. *Vincentii*, j: *F. nucleatum* subsp. *animalis* 7_1, k: *F. nucleatum* subsp. *animalis* D11 (I), l: *F. nucleatum* subsp. *animalis* D11 (II), m: *F. nucleatum* subsp. *animalis* 11_3_2, n: *F. nucleatum* subsp. *polymorphum* 1, o: *F. nucleatum* subsp. *polymorphum* 1.

Docking analysis of *F. nucleatum* FadA proteins with inhibitor peptide showed that all the proteins from virulent bacteria tend to bind with this peptide. Good binding affinity is reflected by the docking energy scores ranging from -136.574 for *F. nucleatum* II to -209.754 for *F. nucleatum* 13_3C (Figure 4, Table 6).

### Docking of FadA protein with COX2

**Figure 5:**
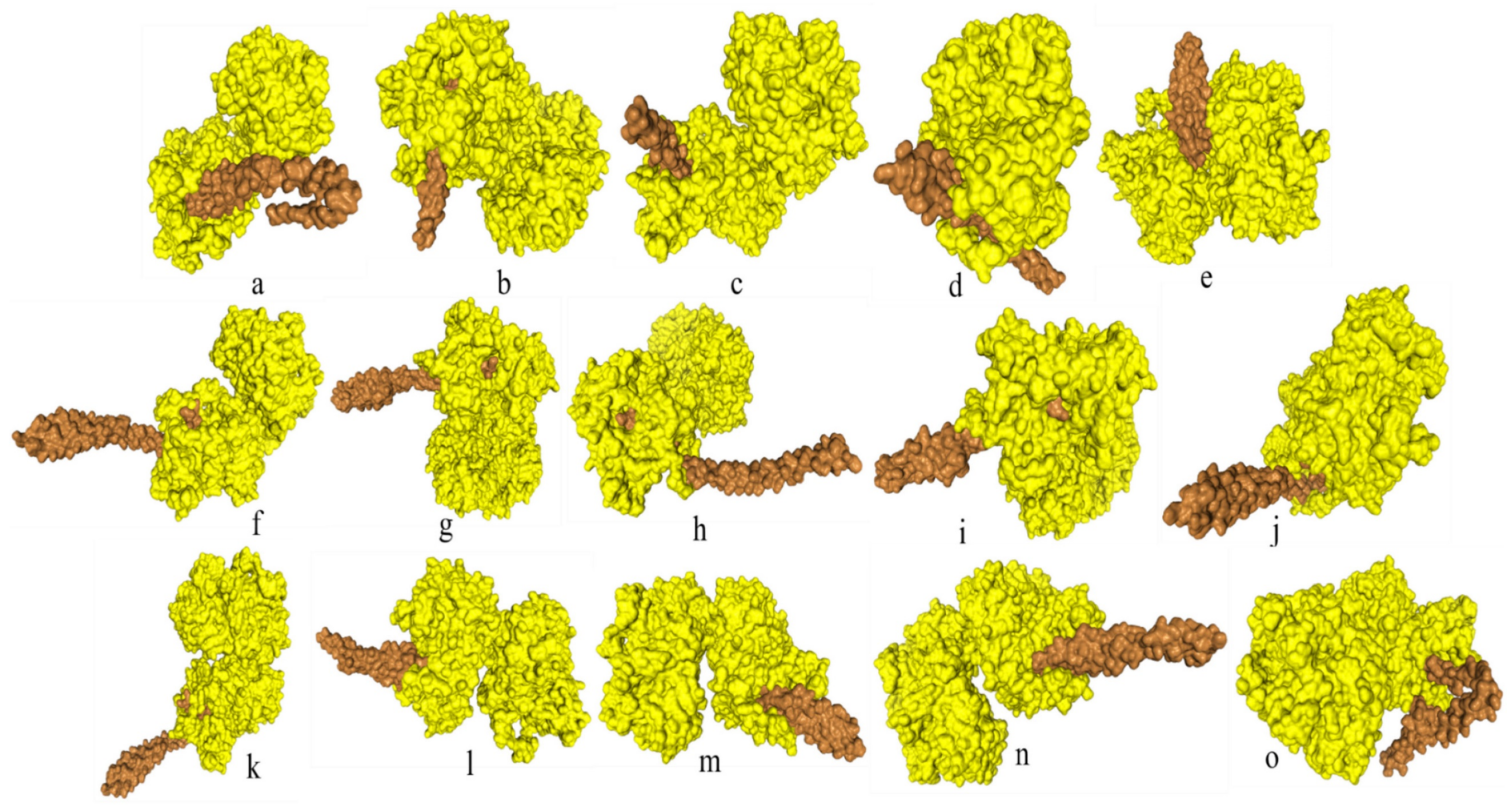
Docking of FadA virulence factor from different strains of *F. nucleatum* with COX2 anti-inflammatory protein FadA and Cox2 are represented in brown and yellow colors, respectively. a: *F. nucleatum* (I), b: *F. nucleatum* (II), c: *F. nucleatum* (III), d: *F. nucleatum* (IV), e: *F. nucleatum* (V), f: *F. nucleatum* (VI), g: *F. nucleatum* 13_3C, h: *F. nucleatum* CTI-5, i: *F. nucleatum* subsp. *Vincentii*, j: *F. nucleatum* subsp. *animalis* 7_1, k: *F. nucleatum* subsp. *animalis* D11 (I), l: *F. nucleatum* subsp. *animalis* D11 (II), m: *F. nucleatum* subsp. *animalis* 11_3_2, n: *F. nucleatum* subsp. *polymorphum* 1, o: *F. nucleatum* subsp. *polymorphum* 1.

Docking analysis of *F. nucleatum* FadA protein with FDA-approved anti-inflammatory drug COX2 revealed a high binding affinity of FadA with this drug with good energy scores ranging from -204.87 to -286.12 (Figure 5).

## Discussion

In present study, FadA protein is compared among the two groups of GIT bacteria. In one group (*F. nucleatum*), this protein causes pathogenicity while in other (*E. cloacae*) it is non-virulent. *F. nucleatum* is reported to occur abundantly in gut of CRC patients. Present research analyzed the distinguishing characteristics of FadA which might contribute to its virulent character in *F. nucleatum*.

FadA has been found virulent in all the strains of *F. nucleatum* documented in the present study as compared to that of *E. cloacae* which is consistent to previous findings 21, 22.

According to the present study,* E. cloacae-associated* FadA protein is localized in the cytoplasm while in six strains of *F. nucleatum,* the localization is different. Marked deviation in half-life was observed in *F. nucleatum* II-IV i.e. 2 min. as compared to > 10 hours in other cases. In cases of *E. cloacae*, an instability index below 40 suggests their poor *in-vitro* stability as compared to *F. nucleatum* 60. An aliphatic index is a measure of protein thermostability 61. In the present work, FadA in the bacteria from GIT of both the normal and diseased individuals was found to be thermostable. GRAVY values indicated the hydrophilic nature of *F. nucleatum* FadA as compared to most of the *E. cloacae* which were hydrophobic with GRAVY score > 0 62. In most cases of *F. nucleatum*, the pI of the FadA virulence factor was observed strongly acidic as compared to slightly acidic pI in cases of *E. cloacae-associated* FadA.

In the present study, healthy individuals associated with bacterial FadA protein have been found to comprise NAD(P)-binding Rossmann-fold and (TIM)-barrel domains. On the other hand, CRC patients' associated bacteria FadA factors were found to consist of four helical up and down bundles, EF-hand and DNA-binding 3-helical bundle. This finding of the helical domain was consistent with literature where the FadA protein monomer of *F. nucleatum* has been reported as alpha-helical which gives rise to a hair-pin-like structure 63.

As far as the 2D configuration is concerned, no beta-sheet content, large number of helix residues and low coil content was observed in *F. nucleatum* FadA protein as compared to that of *E. cloacae*. Eight strains of *F. nucleatum* versus only one strain of *E. cloacae* were found to contain signal peptides. The length of signal peptides ranged between 17-20 residues in *F. nucleatum* protein. Signal peptide comprising eighteen amino acids i.e. MKKFLLLAVLAVSASAFA has been reported in the FadA protein 64.

Differences between 3D configurations of FadA proteins of two groups of bacteria documented in the present study were understandable. *F. nucleatum* FadA contained only a helical configuration, while from *E. cloacae* the globular structure was observed.

The number of tunnels was comparable between FadA proteins of two types of bacteria in the present study, however, only *E. cloacae* (II) exhibited twenty-six tunnels. Different parameters of catalytic pockets were also analyzed. i. e. druggability, length and bottleneck radius. The binding tendency of a catalytic site of protein for a drug is referred to as druggability. FadA in case of *F. nucleatum* subsp.* polymorphum* 2, *F. nucleatum* subsp. *polymorphum* 3 and *E. cloacae* (I), were found to a good therapeutic target drugs with druggability scores closer to 1 65. In all other cases, the FadA was not found to be a druggable protein. Tunnel length and curvature reflected the substrate specificity of the protein. Parameters of tunnel and curvature might be used to reduce FadA activity through the inhibition of its substrate binding and catalysis.

Being virulent, FadA from *F. nucleatum* can be the most appropriate vaccine target against this pathogenic bacterium. Identification of antigenic epitopes could be proven helpful in this regard. In the present study, A-cell epitopes were found to be higher in number in *E. cloacae* i.e. ranging from 18-52 as compared to *F. nucleatum* i.e. 0-20. No epitopes were observed in *F. nucleatum* II and III. Overall, the presence of a variety of epitopes in cases of *F. nucleatum* VI, *F. nucleatum* subsp. *Vincentii*, *F. nucleatum* subsp.* animalis 7_1*, *F. nucleatum* subsp.* animalis* D11 (I) AND (II) and *F. nucleatum* subsp. *animalis* 11_3_2 is consistent with earlier literature reporting antigenic heterogeneity in different strains of *F. nucleatum* 66. A protein containing a large number of A-cell epitopes is capable of stimulating the immune system in the host. So, FadA in *E. cloacae* I, II and III with antigenic peptides 52, 40 and 28, respectively might boost the immune system. The epitopes predicted in virulent FadA from *F. nucleatum* strains might also be used for designing peptide-based vaccine adjuvants after getting insight into their antigenic potential 57.

According to the literature, the FadA gene is highly conserved not only among different strains of *F. nucleatum* but also among other oral species of *Fusobacterium*. Hence, our findings are in agreements with previous reports 64, 67, 69.

Inhibitory peptides might be the promising candidates as immunotherapeutic agents. In the present study, FadA protein from *F. nucleatum* has been docked with an already reported peptide comprising of eleven amino acid residues i.e. ASANWTIQYND. This peptide has been derived from the EC5 protein and is designated as the inhibitory one 8. docking analysis revealed the strong binding affinity of FadA proteins with this peptide. Keeping in view, the involvement of FadA protein in inflammation, this protein has been evaluated for binding tendency with an FDA-approved anti-inflammatory drug COX2 68. The docking analysis results with energy scores of -224.60 to -286.12 reflected the high binding affinity of FadA virulent factor with COX2. Hence, this drug might be used for successful inhibition of *F. nucleatum-associated* FadA protein.

This study is limited to *in-silico* comparison of FadA protein among the healthy and CRC patients gut bacteria. Its findings are significant because they highlighted the pathogenicity associated properties of protein in *F. nucleatum*. However, validity of these findings should be confirmed in future via experimental studies. For this purpose, fecal samples of healthy and CRC patients will be collected and used for isolation of gut associated bacteria 70. Bacteria will be targeted for FadA protein extraction and analysis.

## Conclusion

Wide range of differences observed in FadA protein not only justifies the virulence associated with *F. nucleatum-*derived FadA but also suggests multiple strategies to inhibit the oncogenic potential of this protein. Present study revealed that due to the high instability index, *F. nucleatum* FadA protein tends to remain stable *in-vitro*, so these proteins can be easily studied and mutated in the laboratory. Tunnels and different attributes of catalytic sites explored might be used to design the drugs targeting FadA virulence factors. Antigenic peptides of virulent FadA proteins might be targeted for peptide-based vaccine designing. Divergence of 2D and 3D structure between virulent and non-virulent FadA protein might help to inactivate the virulency form of this protein through site-directed mutation induction leading to configuration alterations in active regions.

## Figures and Tables

**Table 1:** Virulence status of adhesion virulence factor (FadA) in bacteria documented in present study predicted on the basis of VirulentPred tool

#	Bacterium	Prediction score	Virulent / Non-virulent
Bacteria from CRC patients			
1	*F. nucleatum* I	0.5539	Virulent
2	*F. nucleatum* II	1.0496	Virulent
3	*F. nucleatum* III	1.0588	Virulent
4	*F. nucleatum* IV	1.1012	Virulent
5	*F. nucleatum* V	1.1172	Virulent
6	*F. nucleatum* VI	1.1403	Virulent
7	*F. nucleatum* 13_3C	1.0511	Virulent
8	*F. nucleatum* CTI-5	1.0936	Virulent
9	*F. nucleatum* subsp. *vincentii*	1.1082	Virulent
10	*F. nucleatum* subsp. *animalis* 7_1	1.1398	Virulent
11	*F. nucleatum* subsp. *animalis* D11 (I)	1.1275	Virulent
12	*F. nucleatum* subsp. *animalis* D11 (II)	1.1280	Virulent
13	*F.nucleatum* subsp. *animalis* 11_3_2	1.1111	Virulent
14	*F. nucleatum* subsp. *polymorphum* 1	0.9287	Virulent
15	*F. nucleatum* subsp. *polymorphum* 1	1.1545	Virulent
Bacteria from healthy human			
1	*E. cloacae* I	-1.018	Non-virulent
2	*E. cloacae* II	-1.009	Non-virulent
3	*E. cloacae* III	-0.962	Non-virulent
4	*E. cloacae* IV	-0.979	Non-virulent

**Table 2:** Prediction of physicochemical properties of FadA protein from GIT bacteria of colorectal cancer patients and healthy individuals using ProtParam analysis tool

#	Bacterium	No. of amino acids	Molecular weight	Theoretical pI	Half life (hours)	Instability index	Aliphatic index	Ext. coefficient	GRAVY
Bacteria from CRC patients									
1	*F. nucleatum* I	129	14391.04	4.85	>10 hr	28.47	82.79	7450	-0.577
2	*F. nucleatum* II	47	5111.48	4.06	2 min	40.42	77.45	1490	-0.636
3	*F. nucleatum* III	57	6343.76	4.13	2 min	36.36	75.79	2980	-0.740
4	*F. nucleatum* IV	57	6343.76	4.13	2 min	36.36	75.79	2980	-0.740
5	*F. nucleatum* V	85	10700.26	6.94	>10 hr	46.80	81.41	11460	-1.460
6	*F. nucleatum* VI	132	15735.05	5.20	>10 hr	48.33	88.71	14440	-0.873
7	*F. nucleatum* 13_3C	131	15660.98	5.31	>10 hr	52.95	89.39	14440	-0.878
8	*F. nucleatum* CTI-5	102	12023.82	5.21	>10 hr	49.32	84.22	11460	-0.825
9	*F. nucleatum* subsp. *vincentii*	125	14926.11	5.16	>10 hr	51.94	88.24	14440	-0.890
10	*F. nucleatum* subsp. *animalis* 7_1	134	15931.30	5.20	>10 hr	47.92	88.88	14440	-0.855
11	*F. nucleatum* subsp. *animalis* D11 (I)	128	15249.47	5.31	>10 hr	50.36	81.64	14440	-0.963
12	*F. nucleatum* subsp. *animalis* D11 (II)	103	12663.44	5.28	>10 hr	50.37	82.43	12950	-1.261
13	*F. nucleatum* subsp. *animalis* 11_3_2	83	10338.80	6.00	>10 hr	46.13	83.49	11460	-1.431
14	*F. nucleatum* subsp. *polymorphum* 2	122	14037.03	4.98	>10 hr	43.71	97.62	4470	-0.440
15	*F. nucleatum* subsp*. polymorphum* 3	126	13818.63	4.53	>10 hr	69.24	75.32	1490	-0.498
Bacteria from healthy human									
1	*E. cloacae* I	387	40808.08	6.50	>10 hr	28.84	90.13	15470	0.106
2	*E. cloacae* II	729	79759.34	6.21	>10 hr	32.67	93.68	69330	-0.080
3	*E. cloacae* III	252	26511.52	5.97	>10 hr	28.23	88.41	8480	0.099
4	*E. cloacae* IV	135	14481.69	6.35	>10 hr	33.57	91.11	6990	0.061

pI = isoelectric point, GRAVY = grand average of hydropathicity

**Table 3:** Functional domains predicted of FadA protein from GIT bacteria of colorectal cancer patients and healthy individuals using PFP-FunDSeqE tool

#	Bacterium	Predicted fold type
Bacteria from CRC patients		
1	*F. nucleatum* I	4 helical up and down bundle
2	*F. nucleatum* II	4 helical up and down bundle
3	*F. nucleatum* III	4 helical up and down bundle
4	*F. nucleatum* IV	DNA-binding 3-helical bundle
5	*F. nucleatum* V	EF-hand
6	*F. nucleatum* VI	4-helical cytokines
7	*F. nucleatum* 13_3C	4-helical cytokines
8	*F. nucleatum* CTI-5	4 helical up and down bundle
9	*F. nucleatum* subsp. *vincentii*	4 helical up and down bundle
10	*F. nucleatum* subsp. *animalis* 7_1	4 helical up and down bundle
11	*F. nucleatum* subsp. *animalis* D11 (I)	4-helical cytokines
12	*F. nucleatum* subsp. *animalis* D11 (II)	DNA-binding 3-helical bundle
13	*F. nucleatum* subsp. *animalis* 11_3_2	EF-hand
14	*F. nucleatum* subsp. *polymorphum* 2	DNA-binding 3-helical bundle
15	*F. nucleatum* subsp. *polymorphum* 3	DNA-binding 3-helical bundle
Bacteria from healthy individuals		
1	*E. cloacae* I	NAD(P)-binding Rossmann-fold
2	*E. cloacae* II	(TIM)-barrel
3	*E. cloacae* III	NAD(P)-binding Rossmann-fold
4	*E. cloacae* IV	NAD(P)-binding Rossmann-fold

**Table 4:** Prediction of secondary structure of FadA protein in present study bacteria

#	Bacterium	Helix % (residues)	Beta sheet % (residues)	Coil content % (residues)	Signal peptide % (residues)
Bacteria from CRC patients					
1	*F. nucleatum* I	95 (123)	0 (0)	5 (6)	14 (18)
2	*F. nucleatum* II	87 (41)	0 (0)	13 (6)	0 (0)
3	*F. nucleatum* III	89 (51)	0 (0)	11 (6)	0 (0)
4	*F. nucleatum* IV	94 (103)	0 (0)	6 (7)	0 (0)
5	*F. nucleatum* V	92 (78)	0 (0)	8 (7)	0 (0)
6	*F. nucleatum* VI	93 (123)	0 (0)	7 (9)	14 (19)
7	*F. nucleatum* 13_3C	95 (124)	0 (0)	5 (7)	15 (19)
8	*F. nucleatum* CTI-5	84 (86)	0 (0)	16 (16)	19 (19)
9	*F. nucleatum* subsp. *vincentii*	93 (116)	0 (0)	7 (9)	0 (0)
10	*F. nucleatum* subsp. *animalis* 7_1	92 (123)	0 (0)	8 (11)	14 (19)
11	*F. nucleatum* subsp. *animalis* D11 (I)	89 (114)	0 (0)	11 (14)	15 (19)
12	*F. nucleatum* subsp. *animalis* D11 (II)	94 (97)	0 (0)	6 (6)	0 (0)
13	*F. nucleatum* subsp. *animalis* 11_3_2	94 (78)	0 (0)	6 (5)	0 (0)
14	*F. nucleatum* subsp. *polymorphum* 2	96 (117)	0 (0)	4 (5)	14 (17)
15	*F. nucleatum* subsp. *polymorphum* 3	70 (88)	0 (0)	30 (38)	16 (20)
Bacteria from healthy individuals					
1	*E. cloacae* I	36 (141)	19 (74)	44 (172)	0
2	*E. cloacae* II	56 (405)	12 (86)	33 (238)	0
3	*E. cloacae* III	37 (92)	21 (53)	42 (107)	17 (43)
4	*E. cloacae* IV	30 (41)	19 (26)	50 (68)	0

**Table 5:** Pockets and tunnels identified and tunnels parameters of FadA protein of *F. nucleatum* and *E. cloacae*

Tunnels ID	Bacterium	Pocket score	Pocket Volume	Pocket Druggability	Bottle neck radius [Å]	Length [Å]	Curvature	Throughput
1	*F. nucleatum* I	100	614	0.24	3.4	1.5	1.0	0.96
2					2.7	5.9	1.2	0.90
3					2.2	10.1	1.4	0.83
4					1.8	11.6	1.2	0.79
5					2.1	14.6	1.7	0.78
1	*F. nucleatum* IV	100	699	0.11	2.2	2.0	1.0	0.93
2					1.3	6.2	1.2	0.78
3					1.0	11.3	1.3	0.57
1	*F. nucleatum* V	100	470	0.03	2.4	1.5	1.0	0.95
2					1.1	6.4	1.3	0.67
3					1.2	11.2	1.3	0.64
4					0.9	19.1	1.3	0.37
1	*F. nucleatum* VI	100	913	0.44	1.9	2.0	1.0	0.93
2					1.1	7.8	1.1	0.65
3					0.9	14.7	1.7	0.41
4					0.9	17.1	1.6	0.38
1	*F. nucleatum* 13_3C	100	769	0.06	2.1	1.4	1.0	0.95
2					1.0	13.2	1.3	0.43
3					1.0	17.3	1.5	0.43
1	*F. nucleatum* CTI-5	100	906	0.21	1.6	2.4	1.0	0.87
1	*F. nucleatum* subsp. *vincentii*	100	788	0.61	1.7	1.4	1.0	0.90
2					1.6	7.7	1.4	0.78
3					1.0	13.1	1.4	0.41
4					0.9	16.4	2.1	0.29
1	*F. nucleatum* subsp. *animalis* 7_1	100	882	0.26	2.1	2.0	1.0	0.91
2					1.5	3.9	1.0	0.83
3					1.0	12.6	1.6	0.44
1	*F. nucleatum* subsp. *animalis* D11 (I)	100	748	0.68	1.4	4.4	1.1	0.83
2					1.6	4.8	1.0	0.83
3					1.5	13.1	1.3	0.66
1	*F. nucleatum* subsp. *animalis* D11 (II)	100	598	0.38	2.2	5.2	1.6	0.91
2					1.4	9.2	1.6	0.82
3					1.2	10.6	1.5	0.74
4					0.9	15.5	1.5	0.40
1	*F. nucleatum* subsp. *animalis* 11_3_2	100	664	0.10	2.3	2.5	1.0	0.94
1	*F. nucleatum* subsp. *polymorphum* 2	100	984	0.89	1.7	2.0	1.1	0.90
2					1.9	7.8	1.2	0.83
3					2.2	8.4	1.1	0.83
4					1.2	10.2	1.3	0.69
1	*F. nucleatum* subsp. *polymorphum* 3	100	418	0.82	2.0	2.9	1.1	0.93
2					1.7	8.5	1.2	0.82
3					1.5	11.2	1.1	0.68
4					1.0	14.1	1.4	0.51
5					0.9	12.6	1.2	0.46
I	*E. cloacae* I	100	1474	0.89	2.5	1.4	1.1	0.94
2					1.5	7.5	1.2	0.77
3					0.9	8.6	1.2	0.65
4					0.9	13.0	1.4	0.53
1	*E. cloacae* II	99	1230	0.25	3.5	7.8	1.1	0.93
2					3.3	18.6	1.3	0.87
3					3.3	31.6	1.4	0.81
4					2.9	25.6	1.4	0.80
5					2.2	19.6	1.5	0.79
6					3.3	43.0	1.9	0.77
7					3.3	48.2	1.9	0.76
8					3.3	57.4	2.2	0.74
9					3.3	64.2	2.0	0.72
10					1.2	17.7	1.5	0.72
11					1.9	79.5	2.9	0.56
12					0.9	17.1	1.2	0.52
13					1.3	28.2	1.5	0.52
14					1.3	33.2	1.2	0.47
15					1.4	79.7	2.0	0.45
16					1.0	79.1	2.4	0.43
17					0.9	63.4	2.2	0.29
18					1.1	89.0	3.2	0.29
19					1.0	72.2	2.2	0.26
20					1.0	74.4	2.3	0.25
21					1.0	35.7	1.3	0.23
22					0.9	97.0	2.1	0.23
23					1.0	74.2	2.4	0.23
24					0.9	100.5	2.1	0.22
25					0.9	101.4	2.1	0.21
26					0.9	117.7	1.8	0.06
1	*E. cloacae* III	100	987	0.63	2.5	1.5	1.0	0.94
2					2.3	8.7	1.4	0.84
3					1.6	8.3	1.2	0.82
4					1.0	6.9	1.3	0.75
5					1.0	5.9	1.0	0.67
6					1.0	19.1	1.6	0.50
7					0.9	13.3	1.5	0.49
1	*E. cloacae* IV	100	824	0.30	3.0	1.0	1.0	0.95
2					1.8	12.2	1.5	0.83
3					1.3	8.9	1.1	0.72
4					1.1	6.9	1.2	0.71

**Table 6:** Energy scores indicating binding affinities of *F. nucleatum* FadA proteins with reported inhibitor peptide, predicted using HPEPDOCK 2.0 and COX2, predicted using HDOCK server

#	Bacterium from CRC patients GIT	Energy score (Inhibitory peptide)	Energy score (COX2)
1	*F. nucleatum* I	-168.848	-263.89
2	*F. nucleatum* II	-136.574	-204.87
3	*F. nucleatum* III	-147.910	-224.60
4	*F. nucleatum* IV	-182.402	-246.10
5	*F. nucleatum* V	-174.946	-251.38
6	*F. nucleatum* VI	-157.704	-257.04
7	*F. nucleatum* 13_3C	-209.754	-242.70
8	*F. nucleatum* CTI-5	-196.507	-263.34
9	*F. nucleatum* subsp. *vincentii*	-188.468	-224.70
10	*F. nucleatum* subsp. *animalis* 7_1	-169.531	-286.12
11	*F. nucleatum* subsp. *animalis* D11 (I)	-172.986	-261.27
12	*F. nucleatum* subsp. *animalis* D11 (II)	-187.876	-258.17
13	*F. nucleatum* subsp. *animalis* 11_3_2	-168.314	-263.60
14	*F. nucleatum* subsp. *polymorphum* 2	-185.352	-231.14
15	*F. nucleatum* subsp. *polymorphum* 3	-154.926	-242.45
